# Draft Genome Sequence of Linfuranone Producer *Microbispora* sp. GMKU 363

**DOI:** 10.1128/genomeA.01471-15

**Published:** 2015-12-10

**Authors:** Hisayuki Komaki, Natsuko Ichikawa, Akira Hosoyama, Nobuyuki Fujita, Arinthip Thamchaipenet, Yasuhiro Igarashi

**Affiliations:** a Biological Resource Center, National Institute of Technology and Evaluation (NBRC), Chiba, Japan; b NBRC, Tokyo, Japan; c Department of Genetics, Faculty of Science, Kasetsart University, Bangkok, Thailand; d Biotechnology Research Center and Department of Biotechnology, Toyama Prefectural University, Toyama, Japan

## Abstract

Here, we report the draft genome sequence of *Microbispora* sp. GMKU 363, a plant-derived actinomycete that produces linfuranone A, a linear polyketide modified with a furanone ring possessing adipocyte differentiation inducing activity. The biosynthetic gene cluster for linfuranone was identified by analyzing polyketide synthase genes in the genome.

## GENOME ANNOUNCEMENT

Endophytic actinomycetes residing in plants are recently recognized as a potential source of a variety of bioactive compounds (1–3). In our screening for novel secondary metabolites from endophytes, a *Microbispora* strain (*Microbispora* sp. GMKU 363) collected from the root of medicinal plant “Lin Ngo Hao” (*Clinacanthus siamensis* Bremek) in Thailand was found to produce a new polyketide linfuranone A. Linfuranone A is a linear polyketide modified with a furanone ring and has adipocyte differentiation inducing activity (4). In order to identify the genes for linfuranone biosynthesis, the genome of *Microbispora* sp. GMKU 363 was sequenced.

*Microbispora* sp. GMKU 363 is preserved as TP-A0892 and NBRC 110472 at Toyama Prefectural University and the NBRC culture collection, respectively. The whole genome of *Microbispora* sp. TP-A0892 monoisolate was read by using a combined strategy of shotgun sequencing with GS FLX+ (Roche; 80.2 Mb sequences, 10.2-fold coverage) and paired-end sequencing with MiSeq (Illumina; 763.5 Mb, 97.2-fold coverage). These reads were assembled using Newbler v2.8 software, and subsequently finished using GenoFinisher software (5), which led to a final assembly of 85 scaffold sequences of >500 bp each. The total size of the assembly was 7,820,463 bp, with a G+C content of 69.6%. Coding sequences were predicted by Prodigal (6). To assess biosynthetic potential for polyketide and nonribosomal peptide compounds, polyketide synthase (PKS) and nonribosomal peptide synthetase (NRPS) gene clusters were analyzed in the same manner previously reported (7)

This genome contains three type I PKS gene clusters but two of them encoded in Scaffold1 and Scaffold8 have only one PKS gene with a single module. In contrast, the PKS gene cluster in Scaffold6 has five PKS genes in which eleven modules are encoded. Based on its domain organization, this gene cluster was deduced to be responsible for linfuranone biosynthesis. The details of gene organization will be reported in a separate paper. In addition, the genome encodes six NRPS gene clusters and one hybrid PKS/NRPS gene cluster. BLAST search suggested that an NRPS (orf560) in Scaffold1 is an indigoidin synthase and an NRPS (orf28) in Scaffold17 synthesizes a siderophore comprising two ornithine and one threonine molecules. However, the remaining four gene clusters (orf272 to orf280 in Scaffold5; orf70 in Scaffold15; orf26 in Scaffold26 and orf56 to orf55 in Scaffold25; orf43 to orf91 in Scaffold5) display no significant sequence similarities to the known gene clusters in public databases. Products from these unknown NRPSs have not been isolated or characterized from strain GMKU 363.

The genome sequence of this strain provides useful information to further explore endophytic *Microbispora* species as a potential source of bioactive secondary metabolites.

### Nucleotide sequence accession numbers.

The draft genome sequence of *Microbispora* sp. GMKU 363 has been deposited in the DDBJ/ENA/GenBank database under the accession number BCBX00000000. The version described in this paper is the first version, BCBX01000000.
